# The Ebola Virus Interferon Antagonist VP24 Undergoes Active Nucleocytoplasmic Trafficking

**DOI:** 10.3390/v13081650

**Published:** 2021-08-19

**Authors:** Angela R. Harrison, Cassandra T. David, Stephen M. Rawlinson, Gregory W. Moseley

**Affiliations:** Department of Microbiology, Biomedicine Discovery Institute, Monash University, Clayton, VIC 3800, Australia; angela.harrison1@monash.edu (A.R.H.); cassandra.david@monash.edu (C.T.D.); stephen.rawlinson@monash.edu (S.M.R.)

**Keywords:** Ebola virus, VP24, interferon antagonist, nuclear transport, nuclear export sequence

## Abstract

Viral interferon (IFN) antagonist proteins mediate evasion of IFN-mediated innate immunity and are often multifunctional, with distinct roles in viral replication. The Ebola virus IFN antagonist VP24 mediates nucleocapsid assembly, and inhibits IFN-activated signaling by preventing nuclear import of STAT1 via competitive binding to nuclear import receptors (karyopherins). Proteins of many viruses, including viruses with cytoplasmic replication cycles, interact with nuclear trafficking machinery to undergo nucleocytoplasmic transport, with key roles in pathogenesis; however, despite established karyopherin interaction, potential nuclear trafficking of VP24 has not been investigated. We find that inhibition of nuclear export pathways or overexpression of VP24-binding karyopherin results in nuclear localization of VP24. Molecular mapping indicates that cytoplasmic localization of VP24 depends on a CRM1-dependent nuclear export sequence at the VP24 C-terminus. Nuclear export is not required for STAT1 antagonism, consistent with competitive karyopherin binding being the principal antagonistic mechanism, while export mediates return of nuclear VP24 to the cytoplasm where replication/nucleocapsid assembly occurs.

## 1. Introduction

Ebola virus (EBOV, species *Zaire ebolavirus*) is a causative agent of outbreaks of Ebola severe hemorrhagic fever. EBOV and other members of the genus *Ebolavirus* belong to the family *Filoviridae*, which also includes Lloviu virus (LLOV; genus *Cuevavirus*) [1], and the human pathogen Marburg virus (MARV; genus *Marburgvirus*). In common with most other viruses of the order *Mononegavirales*, transcription and replication of the filovirus genome is exclusively cytoplasmic [2].

VP24 is one of seven genes typically encoded by filoviruses [2] and was originally designated a secondary matrix protein. However, accumulating evidence indicates roles in genome packaging and the formation, condensation and intracytoplasmic transport of nucleocapsids (e.g., [3,4,5,6]). Consistent with this, VP24 localizes to cytoplasmic inclusion bodies during infection, which are the sites of nucleocapsid formation [6,7]. EBOV VP24 also antagonizes the type I interferon (IFN)-mediated innate antiviral immune response by blocking nuclear accumulation of the IFN-activated transcription factor signal transducer and activator of transcription 1 (STAT1) via competitive binding to specific karyopherin nuclear import receptors responsible for STAT1 transport [8,9,10]. VP24 was also recently shown to inhibit signaling by complexes of STAT3, through mechanisms including competitive karyopherin binding and VP24–STAT3 interaction, enabling antagonism of responses to interleukin-6 family cytokines [11]. Importantly, accumulating evidence points to VP24 as an attractive target in the development of anti-EBOV drugs, including recent advances using compounds to interfere with the interaction of VP24 with cellular nuclear trafficking machinery [12,13,14]. 

All transport between the cytoplasm and nucleus occurs through nuclear pore complexes (NPCs) that are embedded in the otherwise impermeable nuclear envelope [15,16,17]. Proteins/molecules smaller than c. 40–60 kDa can freely diffuse through the NPC, but specific directional transport of protein cargoes is mediated by expression of nuclear localization and nuclear export sequences (NLSs and NESs) that bind to members of the karyopherin family (also known as importins or exportins). Karyopherins mediate energy-dependent translocation of cargo through the NPC [15]; this enables regulable nucleocytoplasmic localization of proteins, and is absolutely required for transport of cargoes larger than the diffusion limit. In the cytoplasm, karyopherin alpha (Kα) adaptor proteins typically recognize NLSs enriched in positively-charged residues. Kαs also bind to karyopherin β, which facilitates movement of the complex through the NPC. Within the nucleus, cargoes containing NESs (commonly a motif of hydrophobic residues) bind to exportins, including the ubiquitously expressed chromosomal maintenance 1 (CRM1), for export to the cytoplasm [15]. Hijacking of nuclear trafficking pathways for import/export of proteins is common among viruses with cytoplasmic replication cycles, and these processes have been linked to pathogenesis [16].

STAT1 does not contain a classical NLS and uses a conformational NLS that is presented on IFN-activated STAT1 dimers to mediate nuclear import, enabling transcriptional activation of IFN-stimulated genes [18]. STAT1 dimers bind to members of the NPI-1 Kα sub-family (Kα1, 5 and 6) at a site distinct from sites bound by other cellular cargoes containing classical NLSs. VP24 binds competitively to this site, inhibiting nuclear import of STAT1 homo- and heterodimers and other cellular cargoes that use the same site [8,9,10,11,19]. Notably, pharmacological inhibition of the VP24–Kα interaction impairs EBOV replication in vitro [13]. Despite extensive characterization of the VP24–Kα interface, the nuclear trafficking profile of VP24 remains unresolved. 

We report that EBOV VP24 can undergo specific trafficking between the nucleus and cytoplasm, involving a C-terminally located NES that enables CRM1-dependent nuclear export. By identifying critical residues in the NES, we find that VP24 nuclear export is not essential for STAT1-antagonist function, consistent with competitive Kα binding as the key mechanism, and so appears to be required due to the multifunctional nature of VP24 that involves cytoplasmic roles in replication, distinct from immune evasion.

## 2. Materials and Methods

### 2.1. Constructs, Cells, Transfections, Drug Treatments

The construct to express the NLS of human cytomegalovirus UL44 fused to GFP was generated by subcloning from pEPI-GFP-UL44_NLS_ [20] into the pEGFP-C1 vector (Takara Bio USA, San Jose, CA, USA) C-terminal to GFP. Constructs to express full-length or truncated EBOV-VP24 fused to GFP or GFP-UL44_NLS_ are described elsewhere [11] or were generated by PCR from pCAGGS-FLAG-VP24 (kindly provided by C. Basler, Georgia State University), and cloning into pEGFP-C1 or pEGFP-C1-UL44_NLS_ C-terminal to GFP/GFP-UL44_NLS_. L243A/F245A/L249A mutations were introduced into VP24 by site-directed PCR mutagenesis. Constructs to express FLAG-tagged Kα1 and EBOV nucleoprotein (NP) were kind gifts from C. Basler (Georgia State University) and G. Marsh (Australian Centre for Disease Preparedness), respectively. Other constructs are described elsewhere [21]. 

COS7 and HEK293T cells were maintained in DMEM supplemented with 10% FCS and GlutaMAX (Thermo Fisher Scientific, Waltham, MA, USA), 5% CO_2_, 37 °C. Transfections used Lipofectamine 2000 and 3000 (Thermo Fisher Scientific, Waltham, MA, USA), according to the manufacturer’s instructions. To inhibit CRM1, cells were treated with 2.8 ng/mL leptomycin B (LMB, obtained from Cell Signaling Technology or a gift from M. Yoshida, RIKEN, Wako, Japan) for 3 h. To activate STAT1 nuclear localization, cells incubated in serum-free DMEM (with or without 2.8 ng/mL LMB, 3 h) were treated with IFN-α (Universal Type I IFN, PBL Assay Science, Piscataway, NJ, USA; 1000 U/mL, 30 min).

### 2.2. Confocal Laser Scanning Microscopy (CLSM)

Transfected cells growing on coverslips were treated with or without LMB and/or IFN-α before imaging live, or following fixation (3.7% formaldehyde; 10 min, room temperature (RT)), permeabilization (90% methanol; 5 min, RT) and immunostaining. Antibodies used were: anti-FLAG (Merck, Kenilworth, NJ, USA, F1804), anti-STAT1 (Cell Signaling Technology, Danvers, MA, USA, 14994), anti-EBOV NP (Absolute Antibody, Oxford, UK, Ab00692-23.0), and anti-mouse or anti-rabbit Alexa Fluor 568 or 647 secondary antibodies (Thermo Fisher Scientific, Waltham, MA, USA). Imaging used a Nikon C1 inverted confocal microscope with 63 X objective. For live-cell imaging, cells were analyzed under phenol-free DMEM using a heated chamber. Digitized images were processed using Fiji software (NIH). To quantify nucleocytoplasmic localization, the ratio of nuclear to cytoplasmic fluorescence, corrected for background fluorescence (Fn/c), was calculated for individual cells, before calculation of the mean Fn/c for *n* > 30 cells [11,21,22,23].

### 2.3. Co-Immunoprecipitation

Transfected cells were lysed for immunoprecipitation using GFP-Trap beads (Chromotek, Planegg, Germany), according to the manufacturer’s instructions. Lysis and wash buffers were supplemented with cOmplete Protease Inhibitor Cocktail (Roche, Basel, Switzerland). Lysates and immunoprecipitates were analyzed by SDS-PAGE and immunoblotting using antibodies against FLAG (Merck, Kenilworth, NJ, USA, F1804), GFP (Merck, Kenilworth, NJ, USA, 11814460001), CRM1 (Cell Signaling Technology, Danvers, MA, USA, 46249), Kα1 (Abcam, Cambridge, UK, ab154399) and β-tubulin (Merck, Kenilworth, NJ, USA, T8328), and HRP-conjugated secondary antibodies (Merck, Kenilworth, NJ, USA). Visualization of bands used Western Lightning chemiluminescence reagents (PerkinElmer, Waltham, MA, USA). 

### 2.4. Luciferase Reporter Assays 

Cells were co-transfected with pISRE-Luc (in which Firefly luciferase expression is under the control of a STAT1/2-dependent IFN-sensitive response element (ISRE)-containing promoter) and pRL-TK (transfection control, from which *Renilla* luciferase is constitutively expressed), together with protein expression constructs. Cells were treated 8 h post-transfection with or without IFN-α (1000 U/mL) before lysis 16 h later using Passive Lysis Buffer (Promega, Madison, WI, USA). Firefly and *Renilla* luciferase activity was determined in a dual luciferase assay [11,21,24,25]. The ratio of Firefly to *Renilla* luciferase activity was determined for each condition, and calculated relative to that for control cells treated with IFN-α (relative luciferase activity). Data from 4 independent assays were combined, where each assay result is the mean of three biological replicate samples.

### 2.5. Statistical Analysis

Unpaired two-tailed Student’s *t*-test was performed using Prism software (version 7, GraphPad). 

### 2.6. Sequence Analysis

VP24 protein sequences from *Zaire ebolavirus* (NCBI accession no. AGB56798.1), *Tai Forest ebolavirus* (YP_003815430.1), *Bundibugyo ebolavirus* (YP_003815439.1), *Sudan ebolavirus* (YP_138526.1), *Reston ebolavirus* (NP_690586.1), *Lloviu cuevavirus* (YP_004928142.1) and *Marburg marburgvirus* (ABE27080.1) were aligned using the COBALT constraint-based multiple alignment tool (NIH, NCBI). To identify potential NES sequences, VP24 protein sequences were analyzed using the NetNES 1.1 server http://www.cbs.dtu.dk/services/NetNES/ (accessed on 24 March 2020) [26].

## 3. Results and Discussion

### 3.1. EBOV VP24 Undergoes Nucleocytoplasmic Trafficking

**Figure 1:**
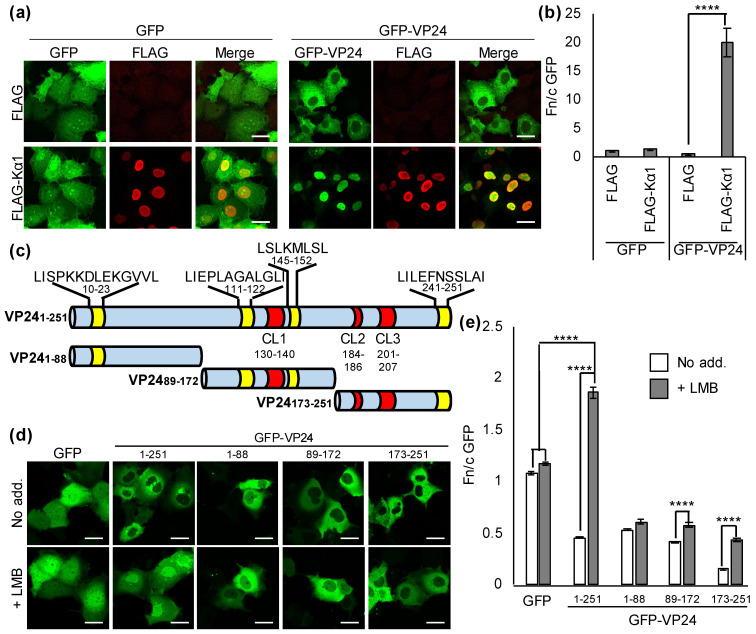
EBOV VP24 undergoes nucleocytoplasmic trafficking. (**a**) COS7 cells co-transfected to express the indicated proteins were fixed 24 h post-transfection before immunofluorescent staining for FLAG (red), and CLSM analysis. Representative images are shown. (**b**) Images such as those shown in (**a**) were analyzed to calculate the Fn/c for GFP (mean ± SEM, *n* ≥ 52 cells for each condition; data from a single assay representative of three independent assays). (**c**) Schematic of VP24 and truncated proteins generated. Potential NESs are indicated in yellow (sequences shown above). Location of residues that interact with Kα5 in the VP24:Kα5 crystal structure (CL1-3) [9] are shown in red. Numbering indicates positions in full-length VP24. (**d**,**e**) COS7 cells transfected to express the indicated proteins were treated 24 h post-transfection with or without LMB before live-cell CLSM (**d**) to determine the Fn/c for GFP (**e**); mean ± SEM; *n* ≥ 31 cells for each condition; data from a single assay representative of three independent assays. ****, *p* < 0.0001; No add., no addition. Scale bars, 30 μm.

EBOV VP24 is largely excluded from the nucleus in infected cells [6,7]. Since VP24 binds to Kαs at a site overlapping the site that mediates active nuclear import of STAT1 [9], it appears likely that cytoplasmic localization involves physical sequestration and/or rapid nuclear export following entry to the nucleus. Karyopherin overexpression is a common approach to examine karyopherin–cargo interactions as it drives high levels of nuclear transport, resulting in nuclear translocation of import cargoes (e.g., [27,28]). Thus, to examine whether VP24/Kα1 complexes can accumulate within the nucleus, we analyzed COS7 cells expressing VP24, with or without co-expression of FLAG-tagged Kα1, by CLSM (Figure 1a), and calculated the nuclear to cytoplasmic fluorescence ratio (Fn/c, Figure 1b) to quantify nucleocytoplasmic localization, as previously described [11,21,22,23].To enable analysis of VP24 localization in both fixed and living cells (below), we expressed VP24 fused to GFP (GFP-VP24), a standard approach used to characterize nuclear trafficking mechanisms of diverse proteins (e.g., [22,23,28,29,30]). GFP fusion also increases the size of VP24 from 24 kDa (below the ~40–60 kDa limit for diffusion through the NPC) to >50 kDa, and so may facilitate identification of active trafficking mechanisms. The localization of GFP-VP24 (below) was consistent with localization reported for VP24 in infected cells [6,7] and in cells expressing HA- or GFP-tagged VP24 [10,31]; GFP-VP24 was also functional for Kα1 binding and antagonism of STAT1 localization/signaling ([11,31] and this study, below), indicating that GFP fusion is well tolerated by the protein.

Immunostained FLAG-Kα1 was strongly nuclear (as expected [32]), irrespective of VP24 expression, and Kα1 expression had no apparent effect on the localization of a GFP control, consistent with the lack of trafficking sequences in this protein. GFP-VP24 could be detected in the nucleus and cytoplasm in cells co-expressing a FLAG control, but localized predominantly to the cytoplasm (Figure 1a,b). However, in cells co-expressing FLAG-Kα1, GFP-VP24 translocated into the nucleus and co-localized with FLAG-Kα1 (Figure 1a,b). This is consistent with the reported interaction of Kα1 with VP24 [8], which we confirmed in co-immunoprecipitation assays (Figure S1), where interaction with overexpressed Kα1 drives VP24 nuclear import. Thus, GFP-VP24 can localize into the nucleus in complexes with Kα1, suggesting that cytoplasmic localization, which is required for roles in nucleocapsid assembly/condensation [3,4,5,6], involves active nuclear export. 

CRM1 is a ubiquitously expressed exportin that mediates nuclear export of many cellular and viral protein cargoes [15,16]. To assess the potential role of the CRM1 nuclear export pathway in VP24 localization, we examined the effect of LMB, an inhibitor of CRM1 [22,23,29] on full-length VP24 (residues 1–251, Figure 1c) using live-cell CLSM (Figure 1d,e). As expected, GFP, which at 27 kDa is below the diffusion limit of the NPC, and lacks NLSs or NESs, was diffusely localized between the cytoplasm and nucleus, with negligible effect of LMB. GFP-VP24_1-251_ was predominantly cytoplasmic at steady state, confirming consistent localization in living (Figure 1d,e) and fixed (Figure 1a,b, above) cells, but re-localized to the nucleus following LMB treatment of the transfected cells (>4 fold increase in Fn/c), resulting in an Fn/c higher than that for GFP alone (Figure 1e), indicative of accumulation. Thus, VP24 localization appears to be dynamic, involving nuclear entry and rapid nuclear export by CRM1. While clear nuclear accumulation of VP24_1-251_ was observed following LMB treatment (Figure 1d,e), the extent of nuclear localization was lower than that observed in cells following co-expression of FLAG-Kα1 (Figure 1a,b); this is consistent with the fact that in the experiments in Figure 1d,e, nuclear import of VP24 is mediated by endogenous Kαs, including Kα1, while in Figure 1a,b, the overexpression of Kα1 will significantly increase cargo-Kα1 interaction and nuclear import.

### 3.2. The VP24 C-Terminus Contains a NES

CRM1 mediates nuclear export of cargoes containing NESs typically conforming to a motif of hydrophobic residues (L-X_(2-3)_-L-X_(2-3)_-L-X-L, where L is L, V, I, F or M, and X is any amino acid [15]). Manual inspection of the VP24 sequence and analysis using the NetNES prediction server [26] identified four potential CRM1-dependent NESs (Figure 1c, Figure S2a). To determine which of these is/are responsible for nuclear export, we generated constructs to express truncated GFP-VP24 proteins comprising N-terminal (VP24_1-88_), central (VP24_89-172_) and C-terminal (VP24_173-251_) portions (Figure 1c). The truncations were designed to be of similar length and to avoid disruption of key structural elements (e.g., alpha helices and beta sheets), based on the VP24 crystal structure [9]. Since the GFP-tagged truncated proteins are <40 kDa in size (Figure S3), they are expected to be able to diffuse through the NPC. Despite this, all proteins were predominantly cytoplasmic at steady state (Figure 1d), indicative of cytoplasmic sequestration and/or active nuclear export. Localization of GFP-VP24_1-88_ was largely unaffected by LMB, and LMB produced only a small (≤1.4 fold) increase in Fn/c for GFP-VP24_89-172_ (Figure 1d,e). Thus, localization of these proteins does not appear to involve substantial CRM1-dependent NES activity, indicating that the principal NES of VP24 is not located in the N-terminal or central region. In contrast, a substantial increase (>2 fold) in Fn/c for GFP-VP24_173-251_ was observed following LMB treatment. The Fn/c for VP24_173-251_ was also consistently reduced at steady state compared with the other proteins. Together, these data indicate that the C-terminal part of VP24 contains prominent NES activity. 

Notably, only full-length VP24 accumulated into the nucleus following LMB treatment, with all truncated proteins remaining significantly less nuclear than GFP alone, indicating that the full sequence is required for efficient nuclear accumulation. Three clusters of residues (CL1-3) in VP24 form contacts with Kα (Figure 1c) [9], so Kα interaction is expected to be impaired in the truncated proteins, each of which lacks at least one CL sequence. Thus, to directly confirm that the indicated NES sequence(s) have classical NES activity (i.e., are able to re-localize NLS-containing proteins), we generated constructs to express VP24_89-251_ (which contains all CL sequences (Figure 1c)) as well as VP24_89-172_ or VP24_173-251_ fused to a heterologous classical Kα/Kβ-binding NLS from human cytomegalovirus UL44 protein [20] (Figure S3). 

Fusion of the UL44 NLS to GFP (GFP-UL44_NLS_) results in a modest increase in nuclear accumulation, as expected (Figure S4a,b) [20]. Fusion of GFP-UL44_NLS_ to VP24_89-172_ or VP24_173-251_ markedly reduced nuclear localization, consistent with nuclear export and/or cytoplasmic arrest. LMB induced only a small increase in Fn/c for GFP-UL44_NLS_-VP24_89-172_, suggestive of cytoplasmic retention or nuclear export mediated largely via an alternative mechanism to CRM1-dependent export. However, LMB induced substantial nuclear localization of GFP-UL44_NLS_-VP24_173-251_ (>4.6 fold increase in Fn/c; Figure S4b), clearly exceeding nuclear localization of GFP-VP24_173-251_ (compare Figure S4b and Figure 1e), consistent with a classical CRM1-dependent NES counteracting the activity of the heterologous UL44 NLS. The Fn/c for GFP-VP24_89-251_ was also markedly increased by LMB, but did not attain an Fn/c similar to that of full-length GFP-VP24 (Figure 1e and Figure S4b), consistent with a requirement for the complete protein sequence for efficient nuclear localization. These data support the presence of CRM1-dependent NES activity in VP24, with the principal NES being within VP24_173-251_.

### 3.3. The C-Terminal NES Is the Principal Sequence Mediating VP24 Nuclear Export

To directly assess the contribution of the C-terminal NES to nuclear export of VP24, we disabled the NES motif by site-directed mutagenesis. Residues 241–251 contain a sequence strongly conforming to a NES (Figure 1c), with L243, F245 and L249 having the highest NetNES scores of identified hydrophobic residues (Figure S2a). In silico substitution of these residues to alanine (termed NES mutant, NM) abolished the predicted NES (Figure S2b). 

**Figure 2:**
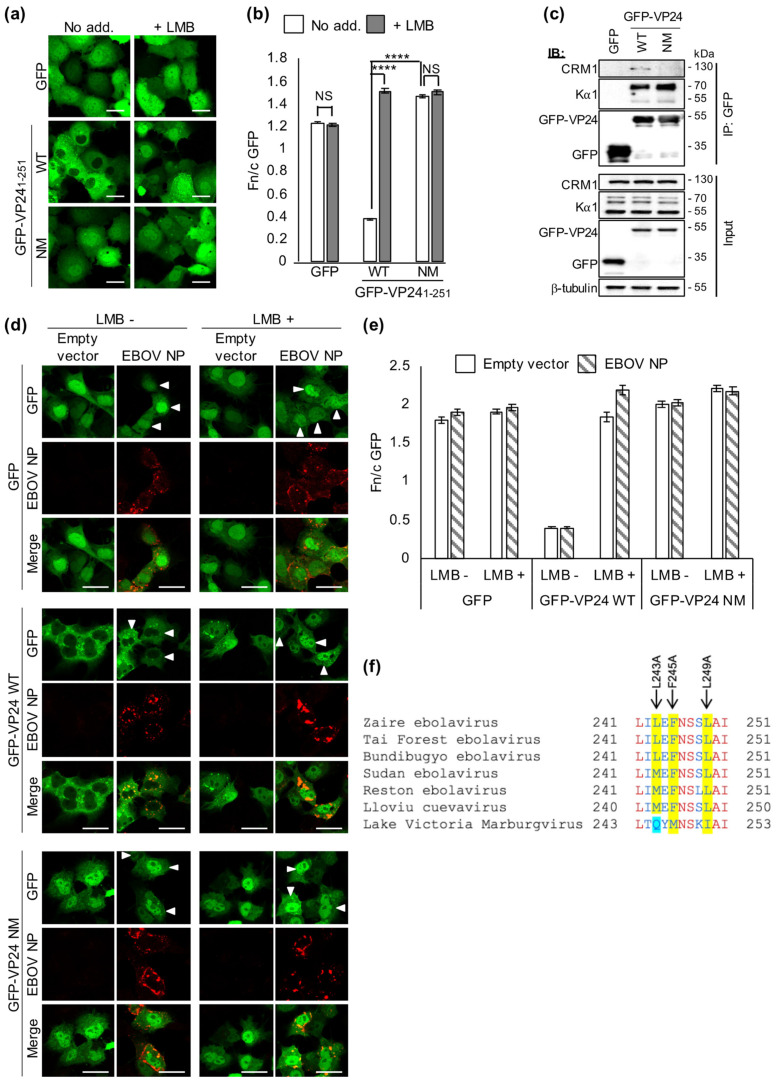
L243, F245 and L249 are critical for VP24 nuclear export. (**a**,**b**) COS7 cells transfected to express the indicated proteins were treated 24 h post-transfection with or without LMB before live-cell CLSM (**a**) to determine the Fn/c for GFP (**b**); mean ± SEM; *n* ≥ 50 cells; data from single assays, representative of three independent assays. (**c**) COS7 cells transfected to express the indicated proteins were lysed 24 h post-transfection before immunoprecipitation for GFP. Lysates (input) and immunoprecipitates (IP) were analyzed by immunoblotting (IB) using antibodies against the indicated proteins. Results are representative of two independent assays. (**d**,**e**) COS7 cells co-transfected to express the indicated proteins were treated with or without LMB before fixation, immunofluorescent staining for NP (red) and CLSM (**d**) to determine the Fn/c for GFP (**e**); mean ± SEM, *n* ≥ 31 cells. Arrowheads indicate cells with detectable NP expression. (**f**) Alignment of the C-terminal 11 residues of VP24. Red and blue font indicate conserved and non-conserved residues, respectively; EBOV VP24 residues implicated in CRM1-dependent nuclear export highlighted in yellow; Q245 of MARV VP24 highlighted in blue. ****, *p* < 0.0001; NS, not significant; No add., no addition; WT, wildtype; NM, NES mutant. Scale bars, 30 μm.

Corresponding mutation of full-length VP24 and analysis of transfected cells indicated significantly enhanced nuclear localization, with an Fn/c equivalent to that for WT VP24 in LMB-treated cells (Figure 2a,b). Furthermore, the mutations ablated effects of LMB. Immunoblotting of cell lysates confirmed comparable expression of GFP-VP24 WT and GFP-VP24 NM at the expected size (Figure 2c, input panel). Thus, L243A/F245A/L249A mutations are sufficient to disable CRM1-dependent nuclear export of VP24, identifying these as critical residues of a novel NES. Similar analysis of GFP-UL44_NLS_-VP24_173-251_ demonstrated that the mutations also largely disabled nuclear export in the truncated protein (Figure S4c,d). Furthermore, immunoprecipitation of GFP-VP24 from COS7 cells detected interaction of CRM1 with VP24 WT but not VP24 NM (Figure 2c), consistent with the presence of a NES in VP24 that is specifically disabled by the mutations. Importantly, both VP24 WT and VP24 NM co-precipitated Kα1, indicating specificity of the NES mutations. 

Since VP24 localizes to cytoplasmic inclusion bodies in infected cells, we considered that other viral factors may effect cytoplasmic sequestration of VP24 and impair nuclear trafficking, such as EBOV NP, an interactor of VP24 and major component of EBOV inclusion bodies [5]. However, co-expression of NP in COS7 cells did not impair nuclear re-localization of VP24 following LMB treatment, and did not affect nuclear localization of VP24 NM (Figure 2d,e). 

This provides evidence of a filovirus protein exploiting specific host trafficking machinery for its own nucleocytoplasmic transport, whereby EBOV VP24 appears to be being actively imported into the nucleus (via interaction with Kα1) and rapidly shuttled back to the cytoplasm via CRM1. During revision of this paper, it was reported that overexpression of FLAG-Kα5 effects nuclear translocation of GFP-VP24 [33], consistent with our data for Kα1 (Figure 1a,b). The acquisition of active nuclear trafficking sequences is consistent with a requirement for highly regulated/dynamic localization. The identified NES was not resolved in VP24 crystal structures [9,34] but C-terminal localization is consistent with exposure and accessibility to CRM1 [9]. Future biophysical analysis of the VP24:CRM1 complex, similar to that performed on the VP24:Kα5 complex [9], will be useful to further define the interface. 

Comparison of VP24 sequences from species of the *Ebolavirus* and *Cuevavirus* genera indicated that L243/F245/L249 are identical or substituted conservatively for hydrophobic residues of the consensus NES motif (L, V, I, F or M; Figure 2f). In contrast, in MARV (genus *Marburgvirus*) the residue corresponding to EBOV L243 is substituted for glutamine (Figure 2f). Consistent with this, a conserved C-terminal NES motif was predicted for all of the *Ebolavirus*/*Cuevavirus* VP24 proteins, but not for MARV VP24, through sequence analysis using NetNES (Figure S2c), suggesting that the latter may lack corresponding NES activity. Interestingly, MARV VP24 is also unique among VP24 proteins of filoviruses in that it does not bind to Kαs [35,36,37]. Kα binding likely mediates VP24 nuclear import, indicated by our finding that FLAG-Kα1 overexpression results in the nuclear translocation of EBOV VP24 (Figure 1a,b) and a previous report that mutation of the EBOV VP24–Kα-binding site inhibits VP24 nuclear localization in fixed/immunostained cells [38]. Thus, it may be hypothesized that active nuclear export is required for filovirus VP24 proteins that bind Kα1 to enable cytoplasmic localization/functions. The lack of Kα binding by MARV VP24 would remove this requirement for active export, potentially accounting for the lack of conservation of key residues of the C-terminal NES. The function or otherwise of the C-terminal NES of MARV VP24, and role of the residue corresponding to EBOV VP24 L243, may be resolved by future research.

### 3.4. EBOV VP24 NES Is Dispensable for IFN/STAT1 Antagonism

**Figure 3:**
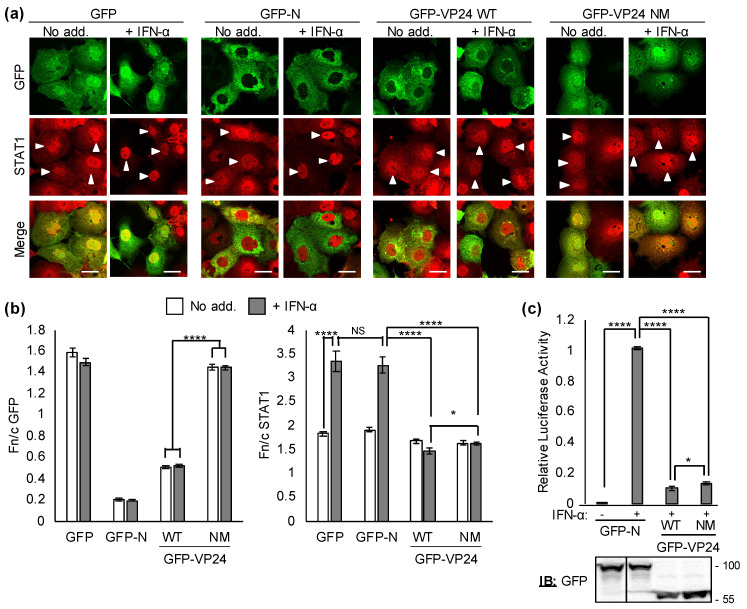
EBOV VP24 NES is dispensable for IFN/STAT1 antagonism. (**a**,**b**) COS7 cells transfected to express the indicated proteins were treated 24 h post-transfection with or without IFN-α (30 min) before fixation, immunofluorescent staining for STAT1 (red) and CLSM (**a**) to determine the Fn/c for GFP and STAT1 (**b**); mean ± SEM, *n* ≥ 36 cells. Arrowheads indicate cells with detectable GFP expression. Scale bars, 30 μm. (**c**) HEK293T cells co-transfected with pISRE-Luc and pRL-TK, and plasmids to express the indicated GFP-fused proteins, were treated 8 h post-transfection with or without IFN-α (16 h) before determination of luciferase activity (mean ± SEM; *n* = 4 independent assays); lower panel: cell lysates used in a representative assay were analyzed by immunoblotting (IB) for GFP. *, *p* < 0.05; ****, *p* < 0.0001; NS, not significant; No add., no addition.

Some viral IFN antagonists use NESs for immune evasion, including through mislocalization of associated STATs [39]. We thus assessed the effect of NES mutations in VP24 on antagonism of IFN/STAT1 using CLSM and an IFN-α/STAT1-dependent luciferase reporter gene assay (Figure 3) [11,21,24,25]. Rabies virus N-protein fused to GFP (GFP-N), which localizes to the cytoplasm and does not affect STAT signaling [29], was included as a standard negative control, as used previously [11,21,25]. GFP-VP24 NM clearly inhibited IFN-α-dependent STAT1 nuclear localization (Figure 3a,b) and reporter gene activity (Figure 3c), to an extent similar to that observed for WT VP24, indicating that the NES mutations do not disrupt VP24 IFN antagonist function. GFP-VP24 also retained IFN/STAT1-antagonist function in LMB-treated cells, despite substantial re-localization of GFP-VP24 to the nucleus (Figure S5). Thus, VP24 nuclear export is not required for inhibition of IFN-α/STAT1, consistent with Kα binding being the major antagonistic mechanism.

## 4. Conclusions

Together, our data indicate that VP24 undergoes specific, regulated nuclear export through interaction with the CRM1 exportin via a novel NES. As VP24 nuclear export does not appear to be required for known functions in immune evasion, it appears that nuclear export activity has been acquired to enable efficient translocation out of the nucleus following Kα1-mediated import, to enable cytoplasmic roles of VP24 in replication [3,4,5,6]. Because VP24 inhibits transcription/genome replication and promotes genome packaging/nucleocapsid condensation [4,5,40], its nucleocytoplasmic trafficking might also contribute to regulation of the transition between replication and assembly, similar to mechanisms proposed for matrix proteins of paramyxoviruses [16]. Notably, paramyxovirus matrix protein nuclear localization has recently been implicated in intranuclear interactions to regulate cellular functions [41,42]. It is therefore intriguing that mass spectrometry analysis identified a large number of proteins in the VP24 interactome with functions related to the nucleus [31], consistent with possible intranuclear roles of VP24.

The multiple roles of EBOV VP24 probably account for the lack of success in generating a VP24-deficient virus [3], but roles in virulence are indicated by the finding that mutations acquired in VP24 during serial passaging in guinea pigs conferred lethality [43]. Notably, the adaptations were not associated with IFN antagonism [43], implying distinct roles for VP24 in pathogenesis. Moreover, a phosphorodiamidate morpholino oligomer that targets VP24 mRNA protects rhesus monkeys against lethal EBOV infection [14], while quercetin was identified as impairing EBOV replication in vitro through disruption of VP24–Kα interaction and VP24-mediated antagonism of IFN/STAT1 [13]. Inhibition of CRM1-mediated nuclear export inhibits replication by diverse viruses, including RNA viruses [44], while mutations impacting the rabies virus P-protein NES correlate with attenuation in vivo [39]. Together, these data highlight the potential for targeting VP24 regulatory mechanisms, including its nuclear export, for anti-EBOV drug design. 

## Data Availability

Data are available in the article and Supplementary Materials.

## Supplementary Materials

The following are available online at https://www.mdpi.com/article/10.3390/v13081650/s1, Figure S1: Kα1 co-precipitates with EBOV VP24. HEK293T cells co-transfected to express GFP or GFP-VP24 with FLAG control or FLAG-Kα1 were lysed 24 h post-transfection before immunoprecipitation for GFP. Lysates (input) and immunoprecipitates (IP) were analyzed by immunoblotting (IB) using antibodies against the indicated proteins. Results are representative of three independent assays. Figure S2: Prediction of NES motifs in VP24 using NetNES. (a) Analysis of full-length and truncated WT EBOV VP24 protein sequences using NetNES identifies four discrete sequences as potential NESs (see Figure 1c); potential NESs were identified by an elevated NES score (pink line) and manual inspection to confirm a NES-like sequence; examples where the NES score does not exceed the threshold (red line) were included if manual inspection indicated a sequence matching a CRM1-dependent NES motif. (b) In silico substitution of residues L243, F245 and L249 for alanine (producing VP24 NM) results in loss of predicted NES function at the VP24 C-terminus. (c) NetNES analysis of the C-terminal 51 residues of VP24 indicates conservation of the C-terminal NES among ebolaviruses and *Lloviu cuevavirus*, but not *Lake Victoria marburgvirus*. NetNES predicts a NES if the NES score (pink line) exceeds the threshold (red line); the NES score is calculated based on Hidden Markov Model (HMM; blue line) and artificial Neural Network (NN; green line) scores [26]. Figure S3: Immunoblotting analysis of truncated VP24 proteins used in Figure 1 and Figure S4. COS7 cells transfected to express the indicated proteins were lysed 24 h post-transfection before analysis of lysates by SDS-PAGE and immunoblotting (IB) using antibodies against the indicated proteins. Data are from single blots with intervening and marker lanes removed. Arrowheads indicate expected molecular weights. Figure S4: EBOV VP24 C-terminal region contains discrete CRM1-dependent NES activity. COS7 cells transfected to express the indicated proteins were treated 24 h post-transfection with or without LMB before live-cell CLSM (a,c) and determination of the Fn/c for GFP (b,d); mean ± SEM; *n* ≥ 40 cells for each condition; data from single assays, representative of two (a,b) or three (c,d) independent assays. **, *p* < 0.01; ****, *p* < 0.0001; NS, not significant; no add., No addition. Scale bars, 30 μm. Figure S5: Inhibition of nuclear export of VP24 does not prevent antagonism of STAT1. (a,b) COS7 cells transfected to express GFP or GFP-VP24 were treated 24 h post-transfection with or without LMB (3 h) and/or IFN-α (30 min) before fixation, immunofluorescent staining for STAT1 (red) and CLSM (a) to determine the Fn/c for GFP and STAT1 (b); mean ± SEM, *n* ≥ 50 cells for each condition. ****, *p* < 0.0001; NS, not significant; No add., no addition. Arrowheads indicate cells with detectable expression of GFP. Scale bars, 30 μm.
