# Supplementary figures and images for: The Ebola Virus Interferon Antagonist VP24 Undergoes Active Nucleocytoplasmic Trafficking
Source: Viruses. 2021 Aug 19;13(8):1650. doi: 10.3390/v13081650 (PMC8402725; doi:10.3390/v13081650)

**IB:**

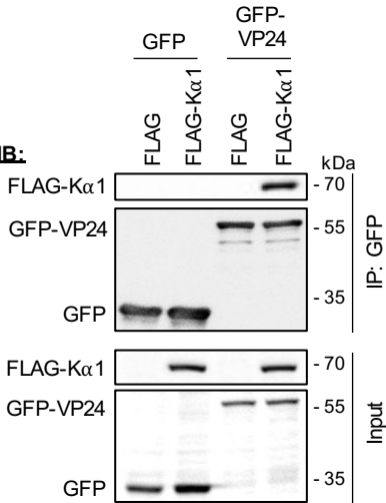

Supplement: Supplementary file 1 [file viruses-13-01650-s001.zip › Figure S1.pdf]

(a)

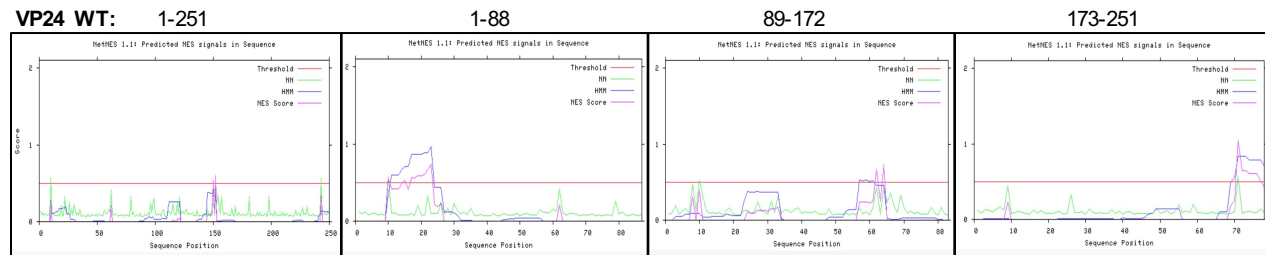

(b)

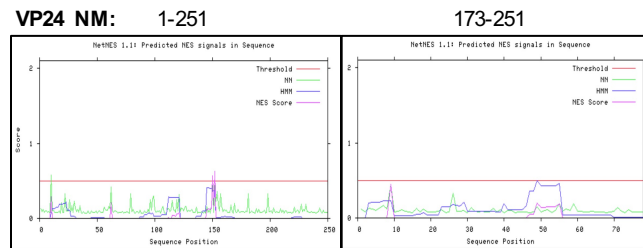

(c)

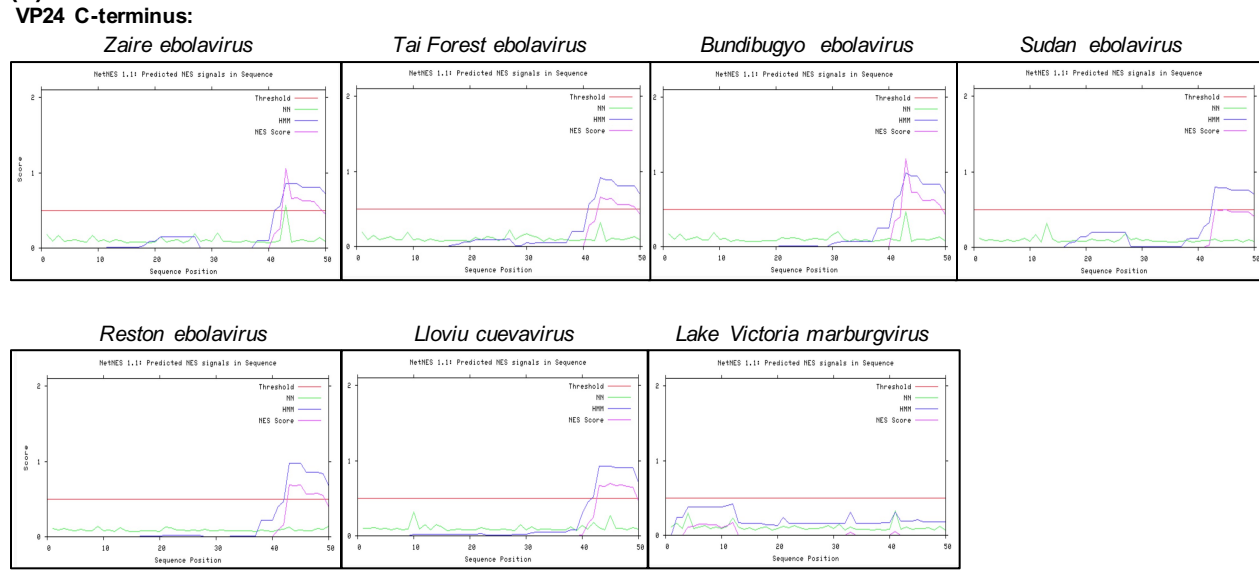

Supplement: Supplementary file 1 [file viruses-13-01650-s001.zip › Figure S2.pdf]

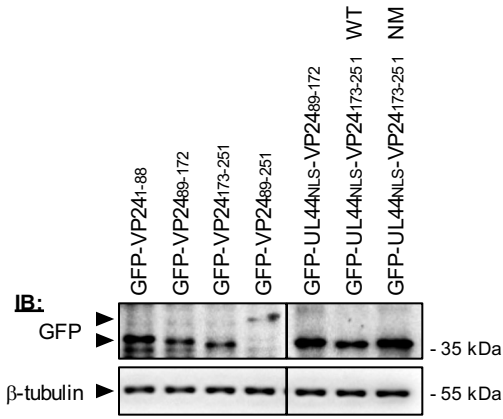

Supplement: Supplementary file 1 [file viruses-13-01650-s001.zip › Figure S3.pdf]

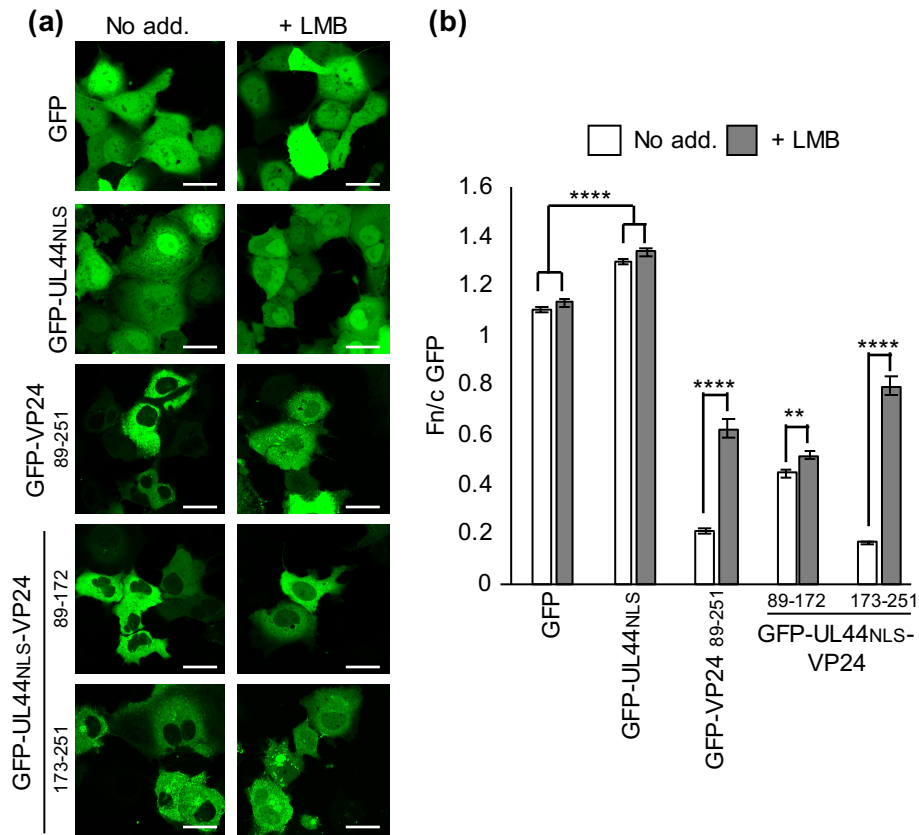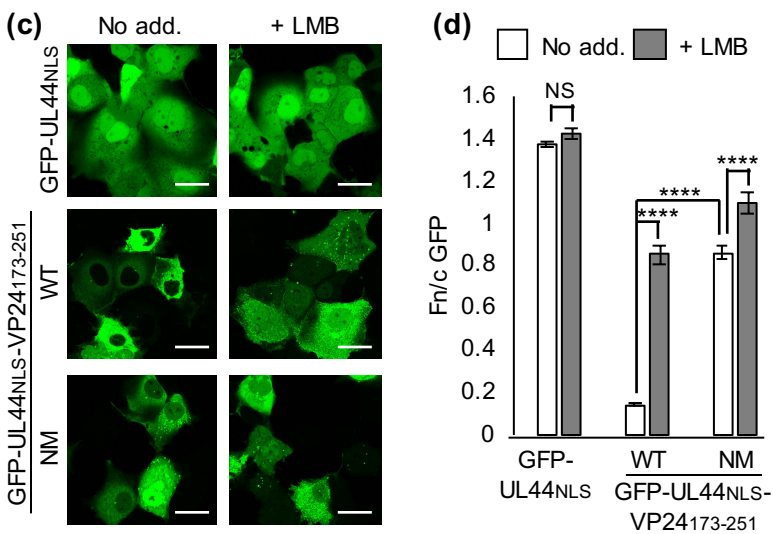

Supplement: Supplementary file 1 [file viruses-13-01650-s001.zip › Figure S4.pdf]

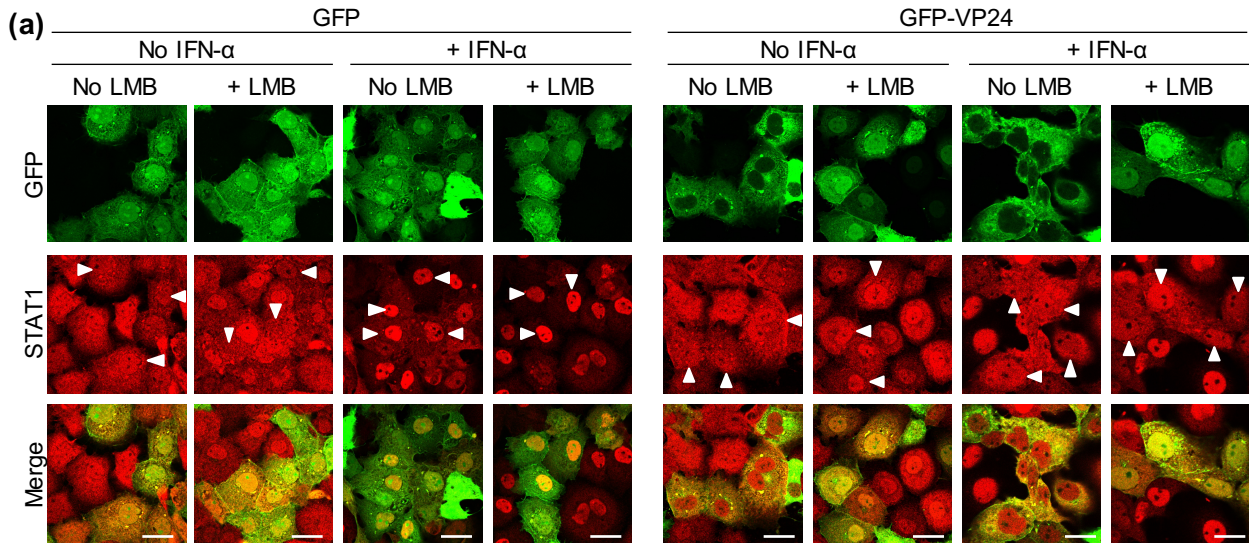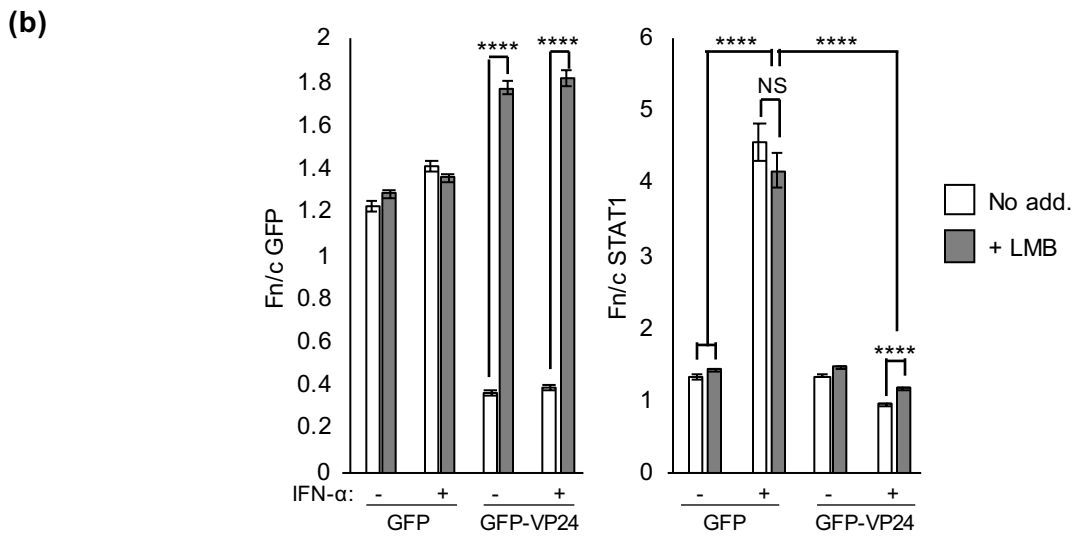

Supplement: Supplementary file 1 [file viruses-13-01650-s001.zip › Figure S5.pdf]
